# The state of telemedicine for persons with Parkinson's disease

**DOI:** 10.1097/WCO.0000000000000953

**Published:** 2021-05-27

**Authors:** Robin van den Bergh, Bastiaan R. Bloem, Marjan J. Meinders, Luc J.W. Evers

**Affiliations:** a Radboud University Medical Center, Donders Institute for Brain, Cognition and Behaviour, Department of Neurology, Center of Expertise for Parkinson & Movement Disorders; b Radboud University Medical Center, Radboud Institute for Health Sciences, Scientific Center for Quality of Healthcare, Nijmegen, The Netherlands

**Keywords:** consultation, monitoring, Parkinson's disease, telemedicine, treatment

## Abstract

**Purpose of review:**

The COVID-pandemic has facilitated the implementation of telemedicine in both clinical practice and research. We highlight recent developments in three promising areas of telemedicine: teleconsultation, telemonitoring, and teletreatment. We illustrate this using Parkinson's disease as a model for other chronic neurological disorders.

**Recent findings:**

*Teleconsultations* can reliably administer parts of the neurological examination remotely, but are typically not useful for establishing a reliable diagnosis. For follow-ups, teleconsultations can provide enhanced comfort and convenience to patients, and provide opportunities for blended and proactive care models. Barriers include technological challenges, limited clinician confidence, and a suboptimal clinician-patient relationship. *Telemonitoring* using wearable sensors and smartphone-based apps can support clinical decision-making, but we lack large-scale randomized controlled trials to prove effectiveness on clinical outcomes. Increasingly many trials are now incorporating telemonitoring as an exploratory outcome, but more work remains needed to demonstrate its clinical meaningfulness. Finding a balance between benefits and burdens for individual patients remains vital. Recent work emphasised the promise of various *teletreatment* solutions, such as remotely adjustable deep brain stimulation parameters, virtual reality enhanced exercise programs, and telephone-based cognitive behavioural therapy. Personal contact remains essential to ascertain adherence to teletreatment.

**Summary:**

The availability of different telemedicine tools for remote consultation, monitoring, and treatment is increasing. Future research should establish whether telemedicine improves outcomes in routine clinical care, and further underpin its merits both as intervention and outcome in research settings.

## INTRODUCTION

Telemedicine is defined as the delivery of healthcare at a distance [1]. Spurred by the COVID-19 pandemic, telemedicine in its various forms has become a widely debated topic. Arguments in favour include the expanded access to multidisciplinary care, reduced travel burden, and convenience of in-home assessments [1,2]. Telemedicine also holds promise to deliver interventions remotely and to measure outcomes at home in the framework of clinical trials [3]. Counterarguments include concerns that implementation of telemedicine might interfere with the intimacy of the clinician-patient relationship, limit diagnostic accuracy, and enlarge inequalities in access to healthcare [4,5,6^▪^].

As the use of telemedicine increases rapidly worldwide to prevent COVID-19 transmission [7], it is crucial to critically delineate the current state of telemedicine. Here, we discuss recent developments in the various fields of telemedicine, covering a period from approximately January 2019 to February 2021. In doing so, we focus on Parkinson's disease (PD) as a model disease for other chronic neurological disorders. Specifically, we will cover three telemedicine approaches: teleconsultation, telemonitoring, and teletreatment. For each area, recent advances are highlighted and placed within a broader context. Pressing limitations and future research avenues will also be discussed. 

**Box 1:**
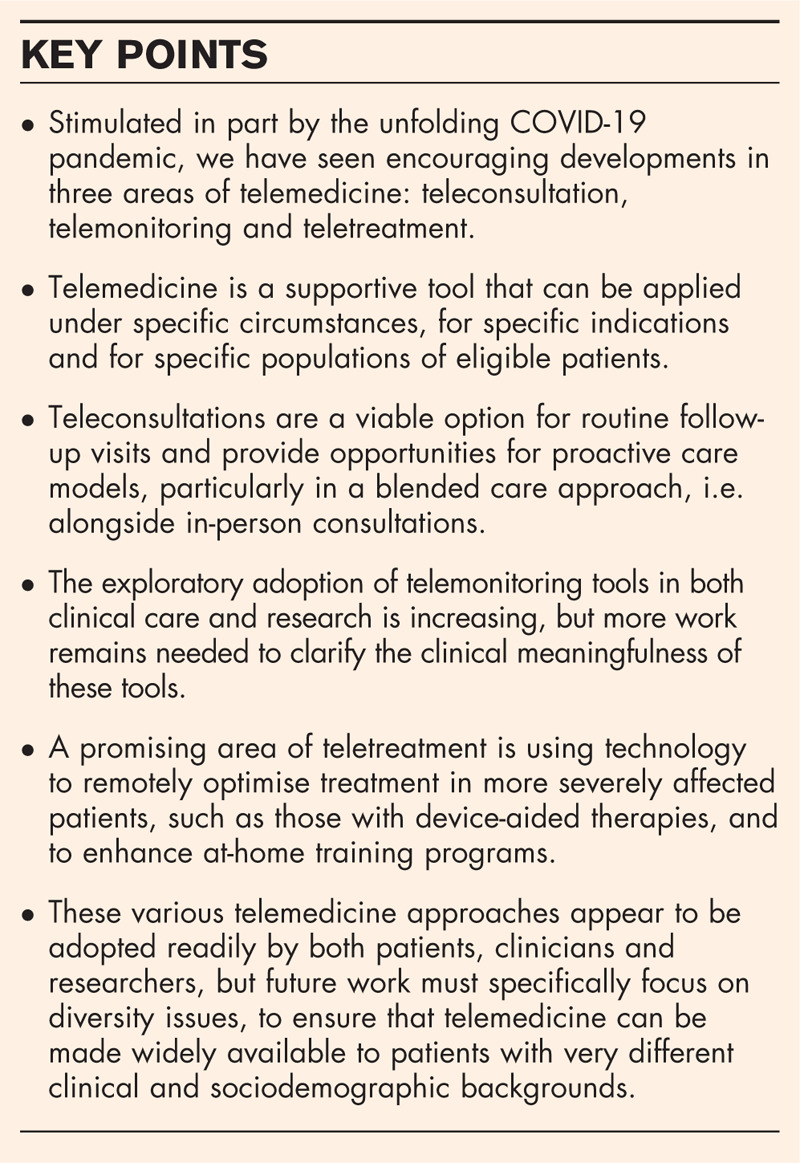
no caption available

## TELECONSULTATION

**FIGURE 1:**
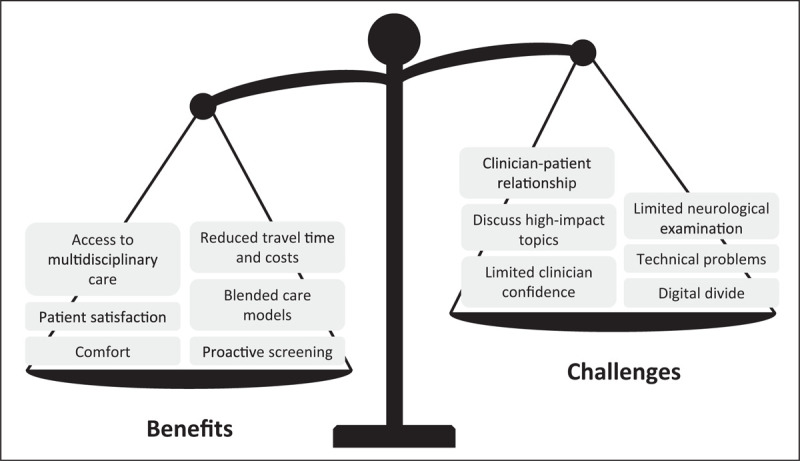
Benefits and challenges of teleconsultations compared to in-person consultations. This figure summarizes the benefits and challenges mentioned in recent studies on teleconsultations. The scale's position reflects the authors’ opinion on the overall readiness of teleconsultations for deployment in clinical practice. *Source*: Original.

Teleconsultation means that the consultation between patient and clinician takes place remotely, e.g. through telephone or video conferencing (for a step-by-step guide, see [8]). In this section, we discuss the reliability and feasibility of remote neurological examinations, the experiences of patients and healthcare providers, and the opportunities for novel care models (Fig. 1).

Parts of neurological examinations can be administered during teleconsultations [9], and this provides comparable results to in-person evaluations for upper limb functioning [10] and evaluation of deep brain stimulation (DBS) candidacy [11^▪^]. However, remote consultations remain limited in their scope because specific assessments – such as rigidity and balance – cannot be performed remotely, and because subtle features such as bradykinesia or tremor are prone to be underdetected by video-based ratings compared to in-person ratings [12]. Indeed, a qualitative study showed that neurologists experienced reduced confidence in their decisions because of these limitations, and additional in-person examinations were often necessary to verify the remote observations [13^▪^]. Therefore, teleconsultations seem only suitable when the medical history or a partial neurological examination is sufficient for the neurologist to adjust the treatment plan. When a diagnosis must be newly established during a very first contact, it remains preferable to see the patient physically to allow for a thorough examination. A caveat here is that in many parts of the world, access to physical care remains restricted, e.g., due to long travel distances and limited provider capacity [14]. Under such circumstances, it is possible to perform at least a part of the neurological examination remotely, which is arguably better than no examination at all.

Overall, persons with PD were satisfied with the delivery of remote consultations [15–18]. The most commonly mentioned advantages include enhanced convenience [15,18,19], greater comfort [15,18], and reduced travel time and costs [11^▪^,13^▪^,^16^,^19^,^20^]. Furthermore, teleconsultations enable enhanced access to specialist care [1,19], especially for patients living in rural areas [20,21] and homebound patients with severe disability requiring palliative care [22]. Common disadvantages mentioned by both persons with RD and clinicians include technical difficulties [13^▪^,^15^,^16^,^19^], lack of hands-on examinations [13^▪^,^19^], and reduced quality of the doctor-patient contact [13^▪^,^19^]. In particular, neurologists had difficulties breaking bad news to patients through telephone or video consultations [13^▪^]. Taken together, teleconsultations can benefit both patients and professionals in specific situations, such as reducing travel burden for stable patients. However, teleconsultations are not suitable when clinicians must address high-impact topics, or when patients themselves prefer an in-person contact [5] or have no access to technology [6^▪^]. Therefore, these experiences of both patients and clinicians suggest that teleconsultations cannot replace all in-person care, but should rather be regarded as an adjunct or additional service that clinicians can use in specific situations [2,4].

Teleconsultations also offer unique possibilities to extend hospital-based care into blended care models, i.e., combining hospital- and home-based care [23]. A remarkable example was implemented in northern Italy where, during the peak of the COVID crisis in early 2020, persons with PD had limited access to in-person care by their own neurologist. These patients were offered remote access to a telenursing service via videoconferencing. Although this Parkinson nurse was a complete newcomer for the patients and could only be seen remotely, the nurse resolved over 60% of incoming requests from patients at a distance, thereby preventing unnecessary travel to the hospital [24]. When more specialized medical care was required, a teleconsultation with a specialist(s) or multidisciplinary team was scheduled during which most issues could be resolved remotely. If needed, subsequent in-person contacts or even hospital admissions were arranged.

Teleconsultations also offer opportunities to provide proactive care, i.e., aiming to identify new medical issues early on so these can be managed timely, thereby preventing avoidable disability and reducing unnecessary costs. An illustrative example is a proactive outreach program that targeted homebound and vulnerable persons with advanced PD and related disorders [25^▪^]. A nurse or social worker proactively called these patients to discuss topics such as home safety, physical and mental wellbeing, medical care provisions, and also lockdown restrictions or scheduling of healthcare appointments. Patients and caregivers reported that the program made them feel safe and supported [25^▪^]. Whether this proactive approach actually avoids medical deterioration and prevents e.g. costly admissions remains to be determined. Similarly, a case report illustrated how intense but completely remotely delivered patient contact could reduce the frequency of falls, which may have prevented fractures or other injuries [26]. The cost-effectiveness of proactive and blended care models must be evaluated in future research.

## TELEMONITORING

**FIGURE 2:**
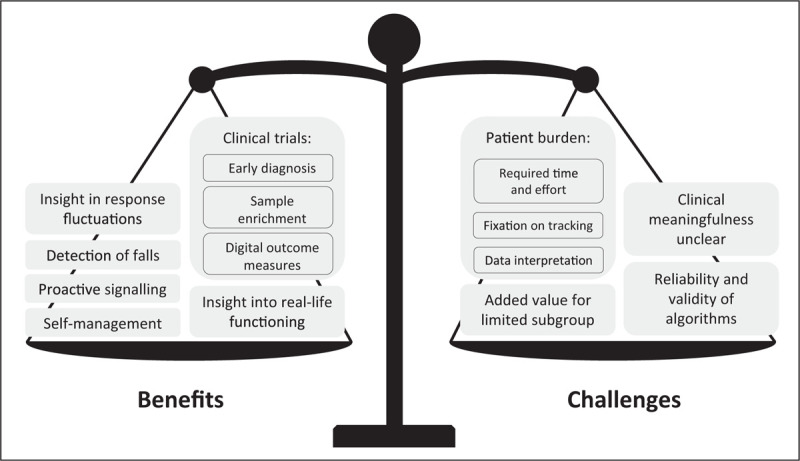
Benefits and challenges of telemonitoring compared to in-hospital measures. This figure summarizes the benefits and challenges mentioned in recent studies on telemonitoring. The scale's position reflects the authors’ opinion on the overall readiness of telemonitoring for deployment in clinical practice and trials. *Source*: Original.

Telemonitoring is the remote gathering of information about a patient which is used to inform healthcare providers (in a clinical setting) or researchers (in the framework of a trial). A wide and expanding spectrum of tools can be used for telemonitoring, including body-worn sensors [27,28], home sensors [29], specific apps for the smartphone [30,31], digital diaries [32], or analysis of common appliances such as computer keyboards [33] (only several selected high-quality references are given here). The promise of remote monitoring is to offer objective, continuous measures of relevant symptoms while patients are at home. This is important because hospital-based assessments can deviate considerably from daily living assessments [34]. Moreover, during in-person visits to the hospital, it remains difficult to reliably ascertain complex fluctuating events (such as response fluctuations to dopaminergic medication), rare events (such as falls [35]) or gradually developing events (such as a slowly progressive decline in physical activities, or disease progression itself) [36]. In this section, we discuss whether telemonitoring tools are ready for use in trials and clinical practice, and what persons with PD think about telemonitoring (Fig. 2).

Perhaps the most immediate application for telemonitoring is its deployment in clinical trials. Recognition is growing that the currently available clinical rating scales may be insufficiently sensitive and accurate to detect meaningful changes in patient functioning; this is particularly problematic in the setting of clinical trials where new experimental interventions are being tested. For that reason, many ongoing and planned studies are incorporating some form of telemonitoring into the overall repertoire of assessments, for now as surrogate, exploratory outcome measures. Recent examples of such studies include a phase 3 study assessing continuous subcutaneous infusion of levodopa/carbidopa [37], and a phase 2 study assessing co-administration of two compounds (CST-103 and CST-107) [38], which both use a wearable sensor to measure at home functioning as secondary outcome. A clear advantage is that telemonitoring, by virtue of the objective and longitudinal assessment in the patient's own home environment, may offer a very sensitive indication of therapeutic benefits. An important challenge is how to interpret such telemonitoring outcomes in terms of their clinical meaningfulness, even when statistically significant [39]. The increasing adoption of telemonitoring in clinical trials, alongside existing measures for patient functioning and quality of life, will help to further refine the reliability and validity of telemonitoring outcomes and support its acceptance by regulatory bodies.

In addition, telemonitoring tools could assist with subject enrolment in clinical trials by enabling early identification of people with PD or prodromal stages of PD. In a 6-year longitudinal study of prodromal individuals, specific gait characteristics such as step velocity and length were predictive of conversion to PD, even when measured as early as up to 4 years prior to the clinical diagnosis [40]. Other technologies suitable for early disease detection encompass touchscreen typing [31] or voice analysis [41]. However, voice studies often relied on high-quality data collected in controlled environments, making it difficult to apply such tools for large-scale screening based on less standardized real-life recordings. One study addressed this issue by collecting telephone-quality voice data from 1483 people with PD and 8300 healthy controls across seven countries [42^▪^]. Although using these real-life data reduced the classification accuracy, this study represents an important step towards analysing data as they would be captured in everyday life.

Incorporating telemonitoring into regular clinical practice faces similar challenges. Recent work indicates that it is feasible and informative to employ telemonitoring tools such as wrist-worn sensors and smartphone applications in clinical practice [43,44,45^▪^,^46^,47^▪^]. However, conclusive evidence of their actual impact on clinical outcomes is lacking. Telemonitoring tools often consist of a dashboard for clinicians that presents the remotely collected data. Pilot studies show positive experiences of clinicians who used such tools in clinical practice. Specifically, the information on symptom severity and medication intake displayed in these dashboards was in line with in-clinic assessments [43], enabled a clinician to make treatment decisions that were comparable to in-person evaluations in most cases [47^▪^], and resulted in more medication adjustments and higher medication doses [45^▪^]. Despite these encouraging initial findings, we lack large-scale randomized controlled trials (RCTs) assessing the effect of such dashboards on clinically relevant outcomes. A recent controlled trial showed improved scores on the Movement Disorders Society Unified Parkinson Disease Rating Scale (MDS-UPDRS) part III and IV in the ON state when the patient's case management was supported by a telemonitoring tool [48^▪^]. However, since no effects were observed on the MDS-UPDRS part II and Parkinson's Disease Questionnaire (PDQ-39), more research is needed to verify whether the benefits translate into an improved patient functioning in daily life. Furthermore, for only few patients, the dashboard provided the clinician with usable information beyond that obtained during the regular clinical evaluation [46,49]. These patients had symptoms that strongly fluctuated [46] or that changed very subtly [49], or who experienced unexpected effects of multiple medications [49]. Therefore, future studies should further identify specific patient populations that may benefit most from telemonitoring tools. Finally, we note that most published work was conducted by groups that also originally developed the monitoring tools under examination. We encourage independent research groups to conduct RCTs to further test the effectiveness of such tools, which will be essential to persuade both the clinical and scientific community about the merits of telemonitoring.

Many persons with PD are motivated to monitor their symptoms, as long as there is a clear goal [50^▪▪^,^51^]. However, a mixed-methods study into the patient's perspectives on self-tracking showed that, even for the most highly motivated patients, it remains necessary to strike a balance between the perceived benefits and the inevitable burden of self-tracking [50^▪▪^]. Specifically, patients reported that self-tracking of e.g. their medication intake or exercise regimes helped them to better understand and manage their PD and to better inform their treating clinician. As a potential burden they mentioned difficulties understanding connections between variables, and getting too fixated on tracking. This balance between benefits and burden could explain the large differences in retention rates between studies. For example, when persons with PD were given (multiple) wearable sensors and were asked to actively provide information using a smartphone-based application, compliance was excellent for up to two weeks [44,52], but decreased steeply after three months [43]. However, when the balance between burden and benefits for patients was improved, e.g., by using only a single tool, by focussing on passive monitoring, and by providing highly personal contact (such as a readily accessible helpdesk), dropout rates could be minimized to 3% after 6 months in one study [49] or even only 1% after 1 year in another [53]. Future research should further improve the balance between benefits and burdens by tailoring the implementation of the monitoring tools to the individual patient's context, measuring only those variables that are relevant and meaningful to both patient and clinician [54^▪^,^55^].

## TELETREATMENT

**FIGURE 3:**
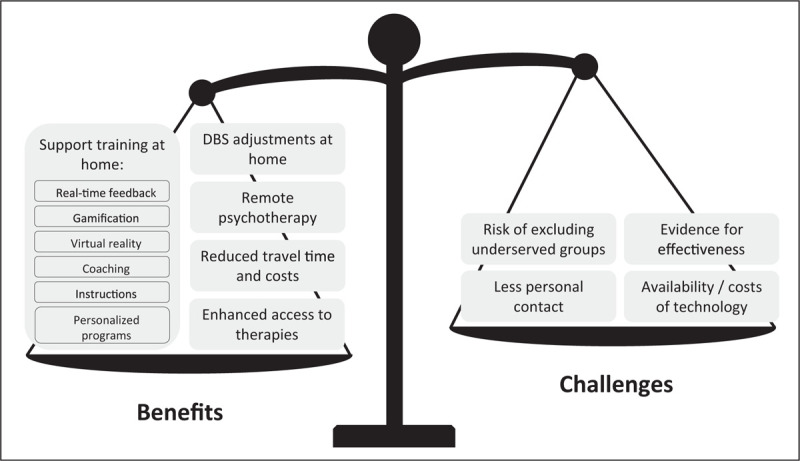
Benefits and challenges of teletreatments compared to in-person treatments. This figure summarizes the benefits and challenges mentioned in recent studies on teletreatments. The scale's position reflects the authors’ opinion on the overall readiness of teletreatments for deployment in clinical practice. *Source*: Original.

The development of technological devices has enabled numerous treatments to be delivered remotely. Here, we review the benefits and challenges of remotely delivered device-assisted therapies, exercise programs, and cognitive behavioural therapy (Fig. 3).

During the COVID-pandemic, parameters for device-assisted therapies such as DBS were successfully adjusted remotely [56^▪^,^57^]. Patients completed self-rated questionnaires about symptom severity and uploaded a video of their motor functioning at home, which were assessed by the hospital-based clinician. Then, whilst video-calling with the patient, the clinician remotely adjusted the parameters of the DBS electrodes during an online therapeutic session. Comparing their condition before and after the parameter adjustments, patients reported a decrease in symptom severity [56^▪^]. The patient satisfaction rates with the remote adjustment sessions were comparable to in-clinic adjustments [56^▪^,^57^]. Although patients reported some difficulty learning how to use the program, these observational results highlight the potential of teletreatment to continue care within the patient's home, even for quite markedly affected patients, and thereby prevent unnecessary travel to the hospital.

For persons with PD, it would be very helpful to be able to perform various nonpharmacological interventions at home, such as physical exercises, speech therapy, or cognitive training. Recent work has shown the feasibility and merits of home-based physical exercise programs which typically included a smartphone-based application or website that showed a personalized training program to patients, with instruction videos explaining which exercises had to be performed and what precautions should be taken [58^▪^,^59^,^60^]. A continued contact with a telecoach using telephone or video calls remained important so patients could ask questions, check whether they were exercising correctly, and could be motivated and supported [59,61]. A double-blind RCT exemplified how technology can further improve home-based physical exercise programs [62^▪▪^]. Specifically, in this study, persons with PD used a home-trainer augmented with virtual reality software and gamified elements to perform aerobic exercises at home, three times a week for six months. The results showed a stabilisation of MDS-UPDRS motor scores and an improvement in VO2 max scores, as compared to an active control group that performed only stretching exercises. Another technology-supported exercise program also appeared to be effective, but only in a more sedentary subgroup of patients [58^▪^]. Therefore, future research efforts should target specific patient groups, e.g., inactive patients, incorporate methods to facilitate personal contact, and continue to develop methods to enhance training programs with technology.

Remote interventions have also been tested for other allied health treatments, such as speech therapy. Specifically, delivering speech therapy remotely can enhance comfort and considerably reduce costs for persons with PD, with only a slight increase in costs for the healthcare system [63]. Technology offers new methods to possibly augment speech therapy, as is illustrated by an innovative RCT study protocol [64]. This study aims to deliver personalized, home-based, online speech therapy to 215 persons with PD. Treatment will be guided online by a speech therapist and, importantly, is supported by a visual feedback application on a smartphone or tablet that shows the patient in real-time whether their pitch is too high or low.

For various chronic neurological diseases, an online rehabilitation program was designed to strengthen both cognitive and physical skills [65]. The program combines virtual reality with a motion sensor so that patients can see their exercises on a screen and interact with them through bodily movements. The prescribed exercises target memory, dual tasking, executive functions, and movement of both upper and lower limbs. Patients received automated feedback on their performance in between exercises, whereas healthcare professionals personalized the content of each training session. Overall, adherence rates were high and patients reported a positive effect on their daily routine and functioning [65].

Finally, two studies delivered teletreatments focused on mental health. One study provided patients with various neurological disorders with a 6-week course that integrated elements from cognitive therapies. Completing the course at home and unsupervised was feasible [66]. An RCT added telephone-based cognitive behavioural therapy to treatment as usual, which led to a stronger reduction in depressive symptoms for persons with PD [67^▪^].

Although these studies offer some careful initial evidence that it is feasible and effective to deliver treatments and support training programs remotely, future research should investigate methods to enlarge the effectiveness and boost the patient experience of these treatments through technology.

## CONCLUSION

A growing body of studies published in the last 2 years has helped to further establish the feasibility and effectiveness of a wide range of different telemedicine tools. Some of the telemedicine tools discussed here are now ready for clinical use in daily practice (e.g. videoconferencing, tools to support exercises), bearing the specific strengths and weaknesses of each approach in mind.

Other tools to remotely monitor and treat patients hold great promise, but require further development and independent evaluations to support their use in clinical practice and research. Diversity should be a specific focus of attention in these new studies, making sure that telemedicine approaches can be made widely available to patients with very different clinical and sociodemographic backgrounds.

Taken together, the time has come to seriously consider telemedicine as one of many useful tools available in our medical and research armamentarium, alongside with established services such as in-person visits to the hospital. Importantly, rather than regarding telemedicine as a panacea for challenges in research and clinical care, we encourage to consider the use of telemedicine as a supportive tool that can be applied under specific instances, for specific indications and for specific populations of eligible patients.

## Acknowledgements

*The Radboudumc Center of Expertise for Parkinson & Movement Disorders was supported by a center of excellence grant of the Parkinson's Foundation.*

### Financial support and sponsorship

*This work was supported by Horizon 2020 (Grant 825785), ZonMw (Grant 91215076), and the Ministry of Economic Affairs by means of the PPP Allowance made available by the Top Sector Life Sciences & Health to stimulate public-private partnerships (Grant TKI-LSH-T2016-LSHM15022).*

### Conflicts of interest

*There are no conflicts of interest.*

## REFERENCES AND RECOMMENDED READING

Papers of particular interest, published within the annual period of review, have been highlighted as:

▪ of special interest
▪▪ of outstanding interest
